# Embracing Ambiguity in the Taxonomic Classification of Microbiome Sequencing Data

**DOI:** 10.3389/fgene.2019.01022

**Published:** 2019-10-17

**Authors:** Nidhi Shah, Jacquelyn S. Meisel, Mihai Pop

**Affiliations:** ^1^Department of Computer Science, University of Maryland, College Park, College Park, MD, United States; ^2^Center for Bioinformatics and Computational Biology, University of Maryland, College Park, College Park, MD, United States; ^3^University of Maryland Institute for Advanced Computer Studies, College Park, MD, United States; ^4^Center for Health-related Informatics and Bioimaging, University of Maryland, College Park, College Park, MD, United States

**Keywords:** microbiome, taxonomy, classification, *16S rRNA* marker gene, high-throughput sequencing

## Abstract

The advent of high throughput sequencing has enabled in-depth characterization of human and environmental microbiomes. Determining the taxonomic origin of microbial sequences is one of the first, and frequently only, analysis performed on microbiome samples. Substantial research has focused on the development of methods for taxonomic annotation, often making trade-offs in computational efficiency and classification accuracy. A side-effect of these efforts has been a reexamination of the bacterial taxonomy itself. Taxonomies developed prior to the genomic revolution captured complex relationships between organisms that went beyond uniform taxonomic levels such as species, genus, and family. Driven in part by the need to simplify computational workflows, the bacterial taxonomies used most commonly today have been regularized to fit within a standard seven taxonomic levels. Consequently, modern analyses of microbial communities are relatively coarse-grained. Few methods make classifications below the genus level, impacting our ability to capture biologically relevant signals. Here, we present ATLAS, a novel strategy for taxonomic annotation that uses significant outliers within database search results to group sequences in the database into partitions. These partitions capture the extent of taxonomic ambiguity within the classification of a sample. The ATLAS pipeline can be found on GitHub [https://github.com/shahnidhi/outlier_in_BLAST_hits]. We demonstrate that ATLAS provides similar annotations to phylogenetic placement methods, but with higher computational efficiency. When applied to human microbiome data, ATLAS is able to identify previously characterized taxonomic groupings, such as those in the class *Clostridia* and the genus *Bacillus*. Furthermore, the majority of partitions identified by ATLAS are at the subgenus level, replacing higher-level annotations with specific groups of species. These more precise partitions improve our detection power in determining differential abundance in microbiome association studies.

## Introduction

The microbiome plays an important role in human and ecological health. One of the first steps in microbial characterization is taxonomic classification. Modern taxonomy was founded in the 1750s by Swedish botanist Carl Linnaeus, who worked to establish a hierarchical classification of organisms based on shared characteristics that were consistent and universally accepted. While the initial taxonomy was able to capture the complex relationships between organisms, maintaining and expanding this taxonomy remain a challenge (Godfray, 2002). In particular, the microbial taxonomy has significantly evolved since the time of Linnaeus, most notably with the advent of next-generation sequencing technologies that enable us to examine microbiota with greater resolution.

Many microbiome studies involve extracting DNA from a microbial community and amplifying and sequencing the *16S rRNA* gene, a gene encoding part of the ribosomal complex. This gene is highly conserved across prokaryotes and can be amplified even from previously unknown organisms. Originally, phylogenetic approaches (Yang and Rannala, 2012) were used to build trees to relate organisms based on how they evolved from each other. These trees were independent of taxonomic annotation and were instead generated directly from sequencing data *via* neighbor-joining (Zhang and Sun, 2008), maximum parsimony (Fitch, 1971; Tamura et al., 2011), maximum likelihood (Stamatakis, 2006), or other methods. Because building a phylogenetic tree is computationally expensive, we often perform taxonomic annotation by searching against a reference database of “known” sequences instead.

There are several limitations to nonphylogenetic approaches. First, it is often impossible to obtain confident species- or even genus-level classifications within samples due to the lack of discriminative power of the sequenced marker gene (Barb et al., 2016). The *16S rRNA* gene contains nine taxonomically discriminating hypervariable regions, however, there is no single hypervariable region of the gene that can distinguish between all species. Additionally, reference databases are not always representative of a sample and are dominated by a small subset of easy to isolate organisms found at higher abundances (Walker et al., 2014). Sequencing data in reference databases is largely biased toward pathogenic microbes and organisms commonly found in developed countries. The organisms found in many studies (e.g., in environmental communities or in developing countries) have no near neighbors in reference databases, making it difficult to assign to them accurate taxonomic labels.

Another problem with modern analysis of microbial communities is the relatively coarse-grained resolution obtained, which limits our ability to capture biologically relevant signals. This stems from the need to simplify computational workflows. Most classification algorithms utilize just seven taxonomic levels and often ignore intermediate taxonomic ranks. This problem is further compounded by errors and missing information in databases, as well as inherent ambiguities in the taxonomic assignment of some sequences. Some taxonomic ambiguity may also arise by taxonomic mislabeling of some entries in the database. Current software tools frequently rely on “most recent common ancestor” (MRCA) strategies to provide an annotation at the most general taxonomic level that encompasses all of the possible annotations of a sequence. As a result, few methods ever make classifications below the genus level, and, frequently, sequences are only classified at the family, class, or even phylum level.

As the number and size of sequencing datasets continues to grow, taxonomic classification methods often make trade-offs between speed and accuracy. Different tools have been developed for taxonomic annotation, using either composition-based, sequence-similarity, or phylogenetic-placement methods (Altschul et al., 1990; Liu et al., 2011; Nguyen et al., 2014; Wood and Salzberg, 2014; Ounit et al., 2015). Composition based and sequence-similarity based approaches are fast and require less computational power, but only work well when the microorganisms in the sample have near neighbors in the database. On the other hand, phylogenetic-placement based methods statistically model the evolutionary processes that generate the query sequences and are computationally expensive, but allow classification even if only distant neighbors are found in databases.

Here, we propose a novel strategy for taxonomic annotation that adequately captures and represents the complexity of the bacterial world, providing more specific and more interpretable characterizations of the composition of microbial communities while also capturing the inherent ambiguity in the classification of sequences. Our strategy is sequence-similarity based and builds upon our recent work on detecting significant “outliers” within database search results (Shah et al., 2018), allowing us to characterize, in a sample-specific manner, the extent of taxonomic ambiguity within the classification. In this work, detecting “outliers” refers to separating the phylogenetically most closely related BLAST matches from matches to sequences from more distantly related organisms. This approach allows us to make assignments at the species level, and even when such assignment is not possible, we may be able to identify the few species within a genus that are the most likely origin of the fragment being analyzed. Such information is particularly relevant in clinical applications, allowing us to distinguish between the pathogenic and nonpathogenic members of the same genus even if the specific species cannot be uniquely identified. It is also important to stress that, by design, our method is conservative - it only provides a classification, even at an intermediate taxonomic level, only when it has high confidence that such a classification is supported by the data. In some cases, particularly for genes such as the*16S rRNA*, which have poor discriminatory power within certain taxonomic group, this will result in sequences being left unclassified, or only classified at high taxonomic levels.

Our method, called “ATLAS-**A**mbiguous **T**axonomy e**L**ucidation by **A**pportionment of **S**equences,” is implemented in Python and released under the open-source MIT license on GitHub [https://github.com/shahnidhi/outlier_in_BLAST_hits]. ATLAS supplements sequence-similarity based approaches with a graph-based approach to identify and group sequences with ambiguous database assignments. We demonstrate that ATLAS yields similar results to phylogenetic methods, but with reduced computational requirements. We use ATLAS to reexamine over 2000 samples from the Human Microbiome Project (HMP) (The Human Microbiome Project Consortium, 2012) and interrogate almost one-thousand stool samples from the Global Enteric Multicenter Study (GEMS) of young children in low-income countries with moderate-to-severe diarrhea (Pop et al., 2014). The HMP dataset provides a large sample size of short-read sequencing data, and the GEMS data is from a population that is underrepresented in our current genomic databases and contains a large proportion of uncharacterized organisms. In these datasets, we identify partitions matching previously defined groupings of organisms within the *Bacillus* genus and the *Clostridia* class. We also demonstrate that the partitions identified by ATLAS increase the power of differential abundance analyses. Although our results specifically focus on data from *16S rRNA* gene surveys, ATLAS can be used with any marker gene sequencing data to characterize the taxonomic composition of a microbial community and to determine microbiome associations with human and ecological health.

## Materials and Methods

### ATLAS Algorithm Overview

**Figure 1:**
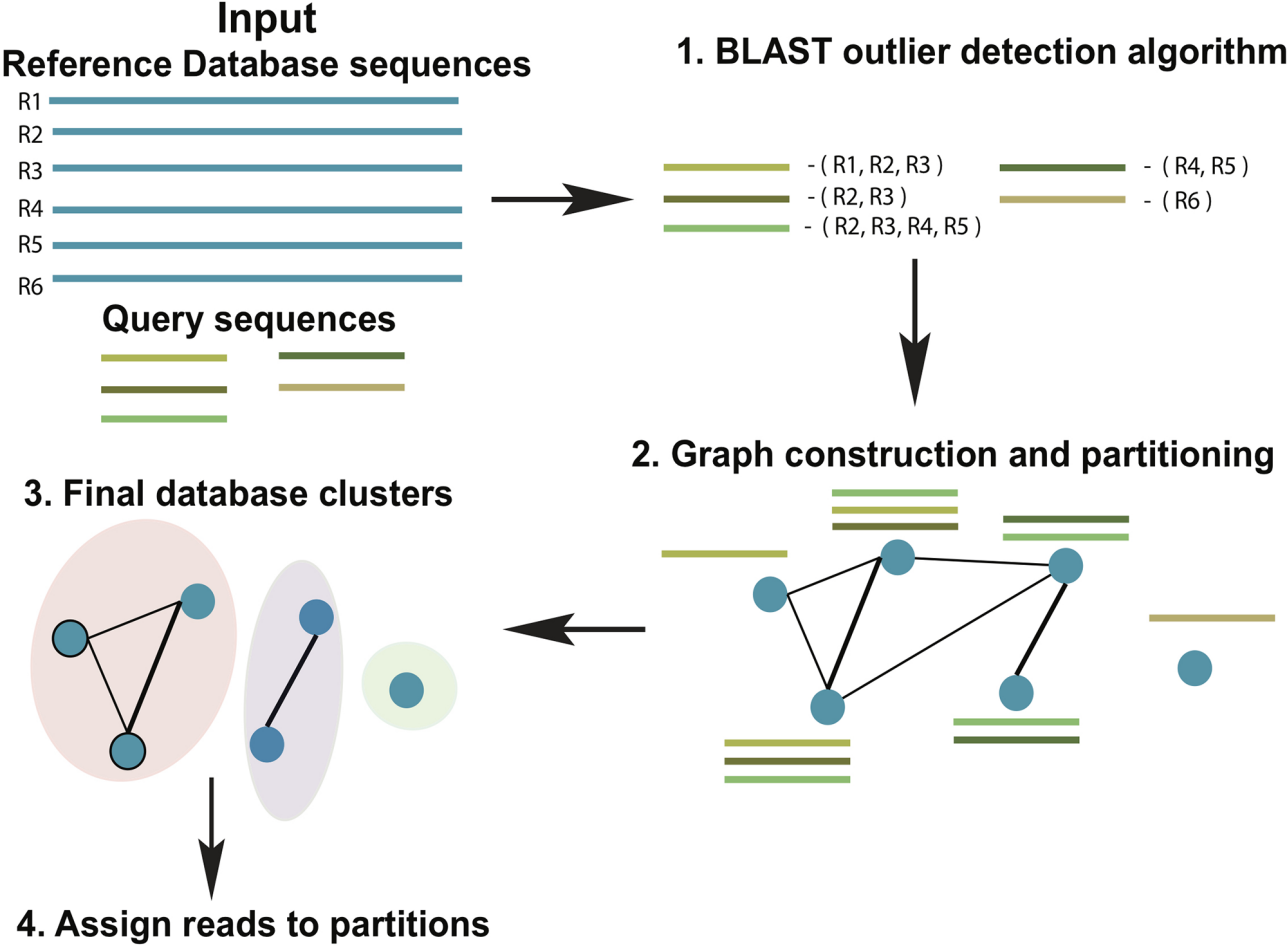
Schematic diagram of the ATLAS pipeline. ATLAS takes in query sequences from a marker gene and searches them against a reference database to identify outlier sequences. It then constructs a graph of database sequences and clusters those that are commonly identified together into partitions.

ATLAS groups sequences into biologically meaningful taxonomic partitions by querying them against a reference database and identifying and clustering significant database hits. ATLAS has two phases (see **Figure 1**): (i) identifying significant database hits for query sequences and (ii) generating database partitions (clusters) that capture the ambiguity in the assignment process.

### Aligning Query Sequences and Identifying Significant Database Hits

ATLAS uses BLAST (Altschul et al., 1990) to align each sequence in an input set of uncharacterized query sequences to sequences in a reference set (using parameters *-outfmt “6 qseqid sseqid pident length mismatch gapopen qstart qend sstart send evalue bitscore qseq sseq”*). The previously published “BLAST outlier detection” algorithm is used to identify significant top BLAST hits for each query sequence (Shah et al., 2018). We refer to these BLAST hits as outliers. In brief, the “BLAST outlier detection” algorithm constructs a multiple sequence alignment of the query sequence and the top BLAST hits from the BLAST-generated pairwise alignments. It then uses the Bayesian integral log odds (BILD) score (Brown et al., 1993; Altschul et al., 2010) to determine whether the multiple alignment can be split into two groups that model the data better than a single group. This process identifies which BLAST hits are significantly associated with the query sequence, without resorting to *ad hoc* cut-offs on percent identity, bit-score, and/or E-value.

### Generating Database Partitions That Capture the Ambiguity in the Assignment Process

**Figure 2:**
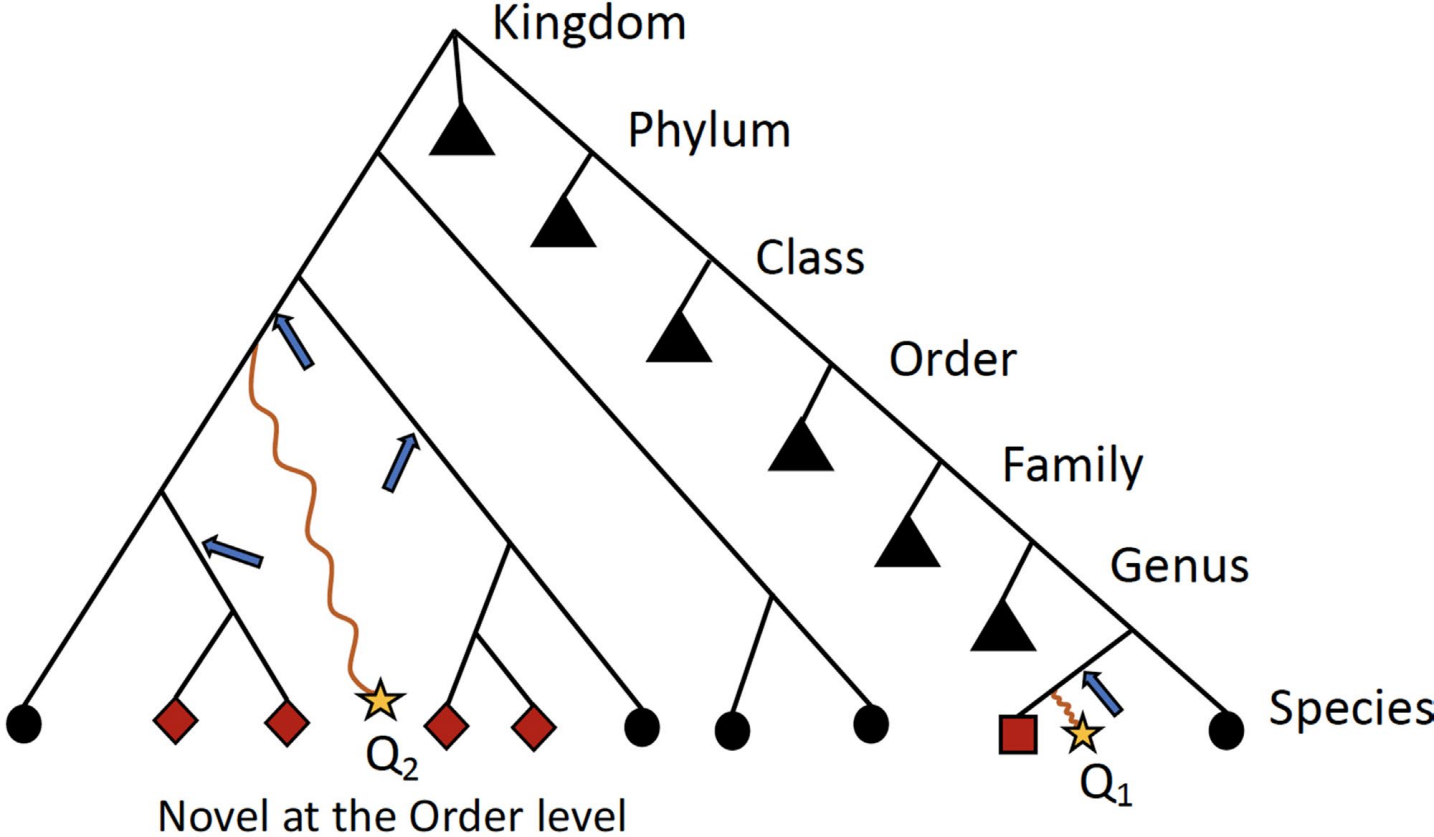
Schematic detailing when ATLAS will provide the greatest improvement to taxonomic annotation. Shown is a simple example of a phylogenetic tree with taxonomic information of reference sequences, where the leaves are actual sequences in the database. When a query sequence (yellow stars) has near neighbors in the reference, such as Q_1_, most algorithms will be able to correctly classify the sequence. However, if a sequence, such as Q_2_, does not have many near neighbors in the database, computationally expensive phylogenetic methods are required for accurate placement (blue arrows) and annotation. ATLAS captures groups (or partitions) of database sequences (red nodes) that are commonly confused during the annotation process and assigns them to the query sequence (square node for Q_1_ and diamond nodes for Q_2_). Black triangles show collapsed portion of the tree. While this schematic is overly simplified and real phylogenies are much more complex, this is illustrating that ATLAS will provide additional information when query sequences do not have near neighbors in the database. This represents ideal cases, where 16S rRNA phylogeny and taxonomic annotations are congruent.

Ambiguity in the taxonomic assignment process occurs for two main reasons. First, the query sequence may not have any near-neighbors in the database, resulting in multiple equally-good hits (neighbors) (**Figure 2**). Second, the query sequence may align to a genomic region that is conserved across distantly related organisms. Our method characterizes this ambiguity in a sample-specific manner, identifying database sequences that are equivalent with respect to their similarity to the set of query sequences.

From all query sequences and their set of related database sequences (outlier set), we construct a confusion graph. The nodes in the graph represent sequences in the database, whereas the edges link nodes that are present together in the outlier set for at least one query sequence. The edges are weighted by the number of query sequences that shares the same nodes (reference database sequences) within the outlier set. Tightly-knit subcommunities in the confusion graph indicate database sequences that are equivalent based on similarity to the set of query sequences, and hence, should be clustered together. To identify these subcommunities, we remove all the low-weight edges (below mean – 2 * std.dev of all edge weights) and identify strong communities in the network using the Louvain community detection algorithm, which optimizes the modularity of the network (Blondel et al., 2008). These subcommunities become the final database partitions (clusters). ATLAS partitions can be singletons (consist of one reference database sequence).

### Assigning Query Sequences to the Partitions

A query sequence is assigned to a database partition if a certain percentage (user-defined, default 50%) of the database sequences in the outlier set belong to the partition. ATLAS does not classify the query sequence if no BLAST outliers can be detected, or the query sequence does not meet these thresholds. The goal of ATLAS is only to classify sequences when it has enough confidence in the taxonomic assignment. Sequences that remain unclassified by ATLAS should be further examined with more sophisticated approaches, such as phylogenetic placement methods. For each query sequence, ATLAS provides a species list based on the reference database sequences included within the assigned partition. To provide a high-level summary of the data and simplify the comparison to other annotation methods, ATLAS also assigns to query sequences the MRCA of all sequences belonging to a partition. These partitions of database sequences attempt to capture the most accurate granularity of taxonomic assignment without relying solely on the main taxonomic levels.

### Comparison to Other Taxonomic Assignment Methods

To benchmark ATLAS with other widely used taxonomic annotation methods, we downloaded TAXXI test and train datasets (sp_ten_16s_v35) from a recent study that benchmarked taxonomic methods for microbiome studies (Edgar, 2018). We compared ATLAS with RDP classifier (Wang et al., 2007), mothur (Schloss et al., 2009), UCLUST (Edgar, 2010), SortMeRNA (Kopylova et al., 2012), and the top BLAST hit. RDP classifier, mothur, and UCLUST were run with 80% confidence threshold. All methods except ATLAS were run *via* QIIME v. 1.9.1 (Caporaso et al., 2010), using the script assign_taxonomy.py. Metrics for method comparison were calculated as previously published (Edgar, 2018).

We also compared ATLAS to the phylogenetic placement method, TIPP. We ran TIPP with the 16S rRNA reference package (rdp_bacteria.refpkg) provided by the authors (https://github.com/tandyw/tipp-reference/releases/download/v2.0.0/tipp.zip). We used the alignment subset size of 100 and the placement subset size of 1,000, and the default values for alignment and placement thresholds.

### Analysis of Samples From the Human Microbiome Project (HMP)

The OTU table and representative sequence FASTA files for the V1-V3 hypervariable region of the *16S rRNA* gene sequenced as part of the Human Microbiome Project (The Human Microbiome Project Consortium, 2012) were downloaded from https://www.hmpdacc.org/HMQCP/. We used the 16S rRNA reference package from TIPP for ATLAS and ran it with default settings. The OTU table was filtered to retain OTUs with at least 20 reads and samples containing at least 1,000 reads.

### Analysis of Samples From the GEMS Study of Diarrheal Disease

A total of 992 samples were analyzed from a previously published study of diarrheal disease in children in low-income countries that sequenced the V1-V2 region of the *16S rRNA* gene (Pop et al., 2014). In this study, moderate-to-severe diarrhea cases were compared to age- and gender-matched healthy controls. Data was downloaded *via* Bioconductor, using the msd16s package. We used the 16S rRNA reference package from TIPP for ATLAS and ran it with default settings. The dataset was filtered to retain only OTUs with at least 20 reads total and found in at least 10% of case or 10% of control samples.

Significantly differentially abundant OTUs were identified between cases and controls using the R package metagenomeSeq (Paulson et al., 2013), accounting for age in months, country, and sample read counts as potential confounding factors. OTUs were also aggregated separately by genus and by partition. Significant findings were reported for features that had fold change or odds ratio exceeding 2 in either cases or controls and a significant statistical association (P < 0.05) after Benjamini-Hochberg correction for multiple testing.

### Analysis of Samples From Bangladeshi Children With Acute Diarrhea

A total of 142 samples were analyzed from a previously published study of acute diarrhea in Bangladeshi children that sequenced the V3-V4 region of the *16S rRNA* gene (Kieser et al., 2018). Fastq files were downloaded from BioProject SRP119744, using the SRA toolkit v. 2.8.2 and processed in QIIME v. 1.9.1. We used the 16S rRNA reference package from TIPP for ATLAS and ran it with default settings, identifying 77 partitions.

## Results

### ATLAS Captures Similar Information as Phylogenetic Placement Algorithms

**Figure 3:**
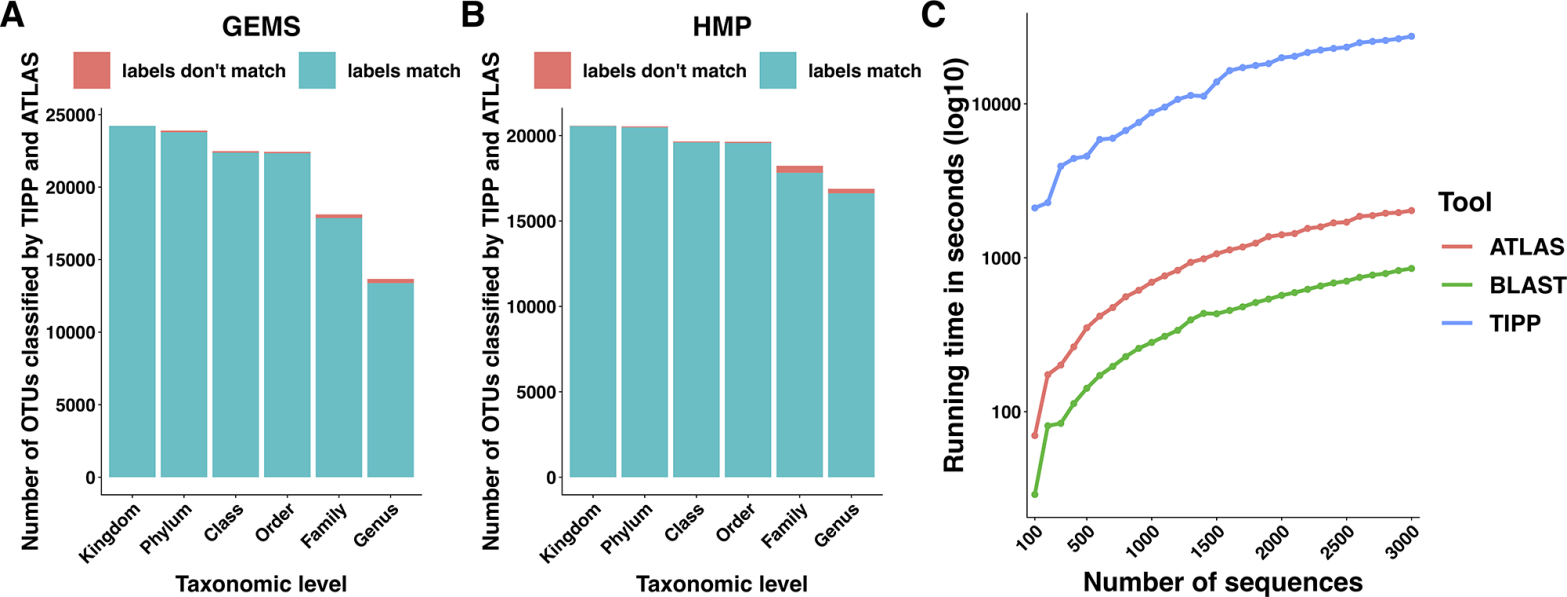
ATLAS generates classifications similar to phylogenetic placement methods at an improved speed. Taxonomic labels assigned by TIPP and ATLAS agree at all taxonomic levels for both **(A)** GEMS and **(B)** HMP datasets. **(C)** The ATLAS pipeline adds minimal post-processing time (in seconds) to standard BLAST analyses, but significantly outperforms TIPP.

We compared the taxonomic assignments generated by ATLAS for the HMP and GEMS datasets to the labels generated by TIPP (Nguyen et al., 2014). Because TIPP relies on a phylogenetic approach for taxonomic annotation, it accounts for evolutionary divergence and, therefore, can more effectively analyze sequences without near neighbors in the database than non-phylogenetic methods. We assume here that the classifications provided by TIPP are most accurate because the ground-truth is not available for real datasets. The taxonomic assignments made by ATLAS and TIPP showed 97% and 98% agreement with TIPP assignments at the genus level for GEMS and HMP datasets, respectively (**Figures 3A, B**). Importantly, when TIPP could confidently assign a species level classification label to a query sequence, but ATLAS could not, the partition assigned by ATLAS for the majority of query sequences contained the species assigned by TIPP (**Table 1**). The algorithm used by TIPP identifies multiple putative placements of a sequence within the backbone tree representing the reference database. In the vast majority of cases, the partitions identified by ATLAS contained the database sequences selected by TIPP (**Supplemental Figure 1**). Compared to TIPP, ATLAS had a lower run time and only added a small overhead to the run time of BLAST (**Figure 3C**).

**Table 1:** Comparison between our approach (ATLAS) and a phylogenetic method (TIPP) examining species level assignments. For most query sequences ATLAS assigned partition contains group of species, as it is often impossible to get species-level resolution. Here, we compare how ATLAS performs when TIPP provides species-level classification.

		GEMS	HMP
**A**.	Number of query sequences classified by TIPP at the species level	13,050	10,086
	Number of query sequences assigned to a partition that contained TIPP’s species	12,847	8,999
**B**.	Number of query sequences classified at species level by ATLAS that match TIPP’s labeling	29	128
	Number of query sequences classified at species level by ATLAS that did not match TIPP’s labeling	0	85
	Number of query sequences classified at species level by ATLAS but not by TIPP	18	36

**(A)** For query sequences where ATLAS partitions do not have a species-level MRCA, the assigned partition contains reference sequences that match TIPP’s assigned species. **(B)** For query sequences where ATLAS partitions do have a species-level MRCA, many of the assigned partitions match TIPP’s classification.

We also compared ATLAS to nonphylogenetic approaches (**Supplemental Figure 2**) on the sp_ten_16s_v35 TAXXI benchmarking dataset where the ground truth is known (Edgar, 2018). Compared to other methods, ATLAS has similar or better overclassification and misclassification rates at all taxonomic levels. However, ATLAS often has a higher underclassification rate, particularly at lower taxonomic ranks. This behavior is intentional as ATLAS is meant to serve as a first-level analysis, followed by more sophisticated approaches (such as phylogenetic placement) for the sequences that cannot be confidently classified through sequence similarity searches.

### Relationship Between ATLAS Partitions and Standard Taxonomic Levels

**Figure 4:**
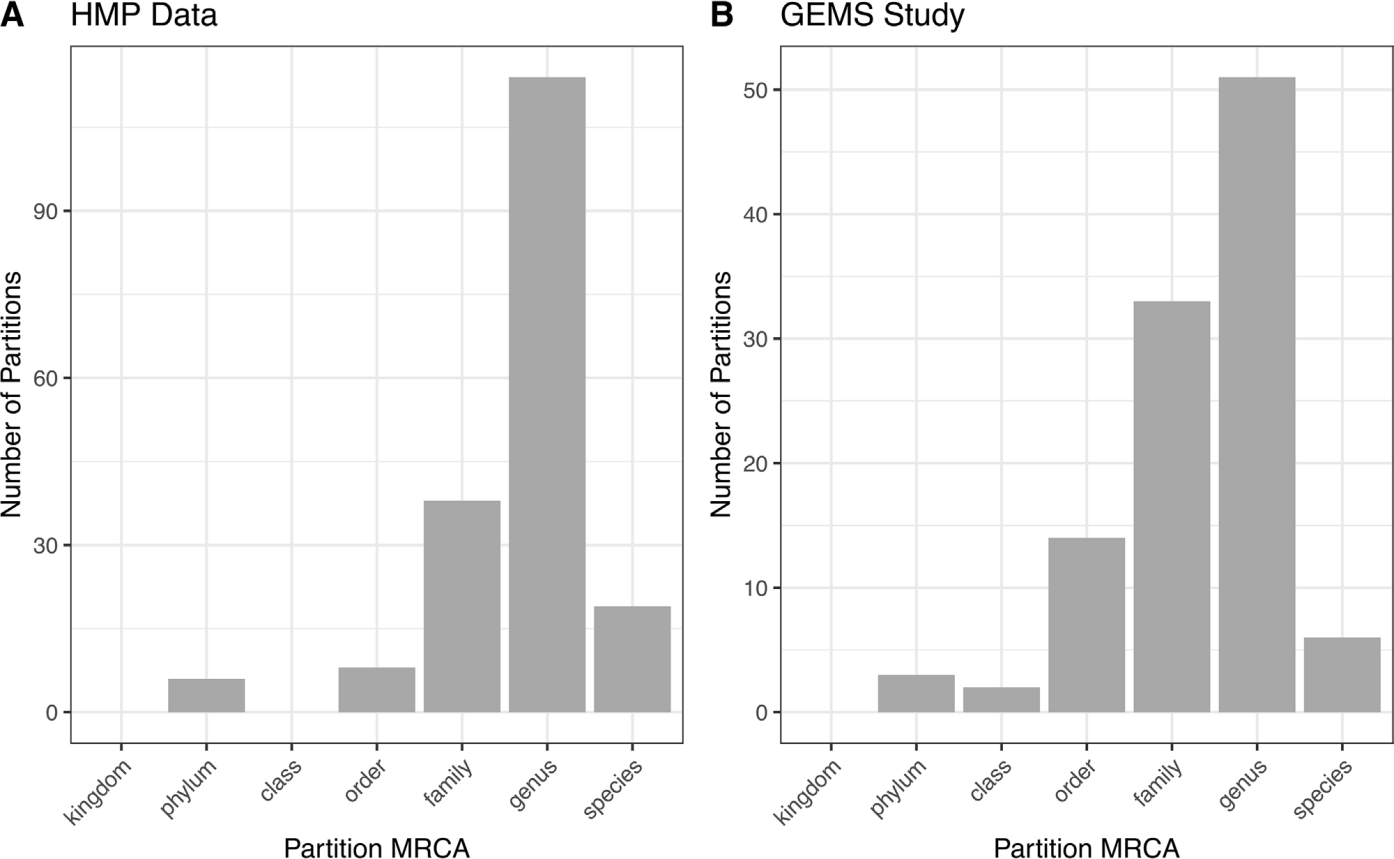
ATLAS partitions for HMP and GEMS data typically capture subgenera information. Most partitions have the most recent common ancestor at the genus level for both **(A)** HMP and **(B)** GEMS datasets.

ATLAS grouped OTU representative sequences into 185 and 109 non-singleton partitions in the HMP and GEMS datasets, respectively (**Table 2**). A large number of these partitions each have an MRCA at the genus level, suggesting that they are capturing sub-genus information (**Figure 4**). Often, there is not enough information encoded in the short *16S rRNA* gene sequence to offer species-level resolution. However, ATLAS is able to group similar species within a genus, providing resolution that is more specific than the genus level. For instance, in the HMP data, ATLAS identified seven partitions belonging to the genus *Bacillus* (**Supplemental Figure 3**). Importantly, reference sequences in partition 156 capture members of the *Bacillus cereus* species group, including *B. cereus*, *B. thuringiensis*, *B. mycoides*, and *B. weihenstephanensis* (Liu et al., 2015). These species have very high sequence similarity and have been shown to play significant roles in human and environmental health (Rasko et al., 2005). ATLAS partition 121 corresponds to the *Bacillus subtilis* group, including species such as *B. subtilis*, *B. licheniformis*, and *B. amyloliquefaciens* (Bhandari et al., 2013). Given the diverse function and pathogenic potential of species within this genus, the distinction of these two groups provides additional benefit to microbiome analyses.

**Table 2:** Number of OTUs and partitions in the HMP and GEMS datasets pre and postfiltering.

	HMP		GEMS		
	OTU	Partition	OTU	Genus	Partition
**Sequencing Technology**	Illumina V1-V3		454 V1-V2		
**Number of Samples Post Filtering**	2,711 180 gut, 1,553 oral, 719 skin, 259 vagina		992 508 Cases, 484 Controls		
**Number of Features Pre-Filtering**	43,140 OTUs	307 partitions and 22,578 non-partitioned OTUs	26,044 OTUs	172 genera	122 partitions and 1,819 non-partitioned OTUs
**Number of Features Post-Filtering**	36,560 OTUs	257 partitions and 17,819 non-partitioned OTUs	10,774 OTUs	149 genera	112 partitions and 924 non-partitioned OTUs

Samples with >1,000 reads were retained for analysis. In the HMP data, features were retained if they had at least 20 total reads or were found in at least 5 samples. In the GEMS data, features were retained if they had at least 20 total reads or were found in at least 10% of case or control samples.

It is important to note that ATLAS partitions are derived purely from sequence similarity; they do not take into consideration any taxonomic or phylogenetic information. Given our incomplete knowledge of microbial diversity and the inherent limitations of 16S rRNA sequences for taxonomic classification, these sub-genus partitions should be further examined and validated.

The percentage of query sequences assigned to partitions spanning multiple genera was 8% for the HMP data and 39% for the GEMS data. Some of these higher-level partition groupings reflect limitations in the hypervariable region of the *16S rRNA* gene sequenced. For instance, in both the HMP and GEMS data, ATLAS identified a single partition spanning the *Enterobacteriaceae* family. While it would be beneficial to distinguish between *Escherichia* and *Shigella* species in the GEMS dataset, the V1-V2 and V1-V3 hypervariable regions of the *16S rRNA* marker gene are insufficient for discrimination (Chakravorty et al., 2007).

Other partitions with higher-level MRCA capture established phylogenetic groupings that span multiple genera. ATLAS was able to capture well-known phylogenetic groupings in the class *Clostridia* (Collins et al., 1994; Johnson and Francis, 1975). In the GEMS data, ATLAS identified 15 partitions comprising sequences from the *Clostridia* class. Of particular note, partition 84 contains *Acetobacterium* species in Clostridial group XV, partition 81 contains members of Clostridial group XI, and Clostridial group I is represented in partitions 5 and 6 (**Supplemental Figure 4**). Clostridial groups encompassed by partitions 0, 81, and 84 contained multiple genera, highlighting the utility of using partitions based on information from the sequences themselves rather than solely relying on modern taxonomic groupings. Interestingly, eight of these partitions were significantly differentially enriched in healthy control samples, supporting the role of *Clostridia* in the maintenance of gut homeostasis (Lopetuso et al., 2013).

### ATLAS Partitions Improve the Power of Microbiome-Disease Association Studies

We explored whether ATLAS partitions could provide improved resolution over OTUs in differential abundance analyses. The original GEMS dataset contains 26,044 OTUs, many of which are not prevalent or abundant enough to provide statistical power for identifying associations between health and disease. Filtering OTUs and partitions according to their abundance and prevalence, we retained just those that contained at least 20 sequences and were found in at least 10% of the samples. Only 10,774 OTUs, comprising just 41% of the sequences in the dataset, were retained, whereas ATLAS partitions retained after filtering contained 25,135 total OTUs, comprising 97% of the sequences in the dataset (**Table 2**).

We identified statistically significantly different features between cases with diarrheal disease and healthy controls (**Table 3**). We performed this analysis separately on (i) OTUs, (ii) OTUs aggregated by genus-level assignments, and (iii) OTUs aggregated by ATLAS partitions. Compared to the OTU analysis, OTUs aggregated at the genus-level generally identified more significant OTUs, but fewer overall significant dataset sequences. This is potentially impacted by the fact that 2,411 OTUs and 899,322 sequences had no assignment at the genus level. OTUs aggregated by ATLAS partitions identified a greater number of significant OTUs and sequences enriched in the control samples. When looking at the 10,774 OTUs included in both the OTU-level and partition-based analyses, the majority agreed on differential abundance results (i.e., they were significant or not significant in both analyses) (**Table 4**). Forty-one percent were significant by the partition analysis, but not by OTU based methods. These OTUs were most likely lower abundant community members that became significant as they were aggregated with similar, more abundant microbiota. The few remaining OTUs were significant at the OTU level but not in our partition-based analyses and generally belonged to low abundance genera (**Supplemental Figure 5**).

**Table 3:** Number of OTUs, genera, and ATLAS partitions that are statistically significantly different between moderate-to-severe diarrheal cases and healthy controls.

	OTU	Genus	Partition
**Significant Features with increased expression in case samples**	679 OTUs (415,257 sequences)	16 genera (892 OTUs, 342,960 sequences)	13 partitions and 71 non-partitioned OTUs (692 OTUs, 189,005 sequences)
**Significant Features with increased expression in control samples**	1,112 OTUs (637,591 sequences)	22 genera (1,626 OTUs, 447,680 sequences)	17 partitions and 108 non-partitioned OTUs (4,917 OTUs, 1,300,544 sequences)
**Non-significant Features**	8,983 OTUs (2,448,992 sequences)	105 genera (5,845 OTUs, 1,811,878 sequences)	77 partitions and 745 non-partitioned OTUs (5,165 OTUs, 2,012,291 sequences)

Features generated from 3,501,840 GEMS dataset sequences were considered differentially abundant if they had a fold change or odds ratio exceeding 2 in either cases or controls and the statistical association was significant (P < 0.05) after Benjamini-Hochberg correction for multiple testing. Singleton partitions have a single OTU mapped to them. Note that when aggregating at the genus level, 2,411 OTUs and 899,322 sequences had no assignment.

**Table 4:** Confusion matrix highlighting the number of shared/unshared statistically significant OTUs and ATLAS partitions.

		OTUs	
		Not Significant	Significant
**Partitions**	**Not Significant**	4,557	608
	**Significant**	4,426	1,183

Features were considered differentially abundant between healthy controls and diarrheal cases if they had a fold change or odds ratio exceeding 2 in either cases or controls and the statistical association was significant (P < 0.05) after Benjamini-Hochberg correction for multiple testing.

We also applied ATLAS to a separate acute diarrhea dataset from children in Bangladesh (Kieser et al., 2018), which used a different hypervariable region of the *16S rRNA* gene, a different sequencing platform, and different downstream analyses. Within this dataset, we also identified sub-genus level partitions (**Supplemental Figure 6A**). Many of the sub-genus level partitions in the Bangladesh dataset were in *Lactobacillus*, *Streptococcus, Helicobacter*, and *Campylobacter*, genera which are commonly associated with diarrheal disease (**Supplemental Figure 6B**).

## Discussion

As DNA sequencing technologies become faster and cheaper, the number of microbiome studies are rapidly increasing. These studies are aimed at both developing a better understanding of the microbial communities inhabiting the world and at characterizing the association between microbiota and health. Accurate taxonomic assignment is a critical requirement for the interpretation of the data generated in such studies. Current approaches for taxonomic annotation fall at two extremes – computationally intensive phylogenetic inference methods that can accurately classify even sequences that are only distantly related to the reference database and fast approaches based on sequence alignment or k-mer analysis that are primarily effective in identifying already characterized sequences. Here, we have described an approach that bridges the two extremes. While it is based on sequence-similarity approach, ATLAS provides a similar level of accuracy as phylogenetic approaches while retaining computational efficiency.

ATLAS identifies the ambiguity in the classification of sequences in a sample-specific manner, thereby obviating the need for removing redundancy from the reference database (a computationally expensive process) and ensuring that the method effectively adapts to the specific parameters of the experiment (e.g., choice of hypervariable region in the *16S rRNA* gene). While ATLAS is intended to replace commonly-used “most recent common ancestor” (MRCA) approaches that are unnecessarily conservative, it can also improve on such techniques. The ATLAS partitions are constructed after examining all the query sequences, and after removing spurious connections between database sequences, thereby eliminating many of the errors that can reduce the taxonomic resolution of the MRCA approach.

We have shown that ATLAS is effective in analyzing real microbiome datasets, where it is able to automatically discover taxonomic groupings that are relevant to the interpretation of the data but that do not match predefined taxonomic levels. Examples include subdivisions of the *Bacillus* genus and Clostridial class homology groups. Our paper describes results generated from *16S rRNA* gene sequencing data, however, the approach is applicable to any other marker gene dataset. Because ATLAS relies on marker gene data, it can only provide a level of resolution matching that of the maker gene itself.

Our analysis of the HMP and GEMS datasets reveals a difference in the level of ambiguity identified by ATLAS; our method was able to better resolve the taxonomy of sequences from the HMP project than that of sequences from the GEMS dataset. This finding is likely due to the relationship between the sequences from the two studies and the data found in the reference database. The GEMS study contains data from children from sub-Saharan Africa and Southeast Asia, sequences that are only distantly related to the reference sequences primarily characterized within Western populations. Our findings support the idea that the choice of database plays a huge role in classification accuracy (Nasko et al., 2018). To ensure an accurate taxonomic annotation, a custom environment-specific database is desirable, and the accuracy of the database sequences and their annotation must be ensured. Studies must also carefully consider and document the choice of database.

The GEMS dataset was generated several years ago using 454 sequencing technology with high-insertion-deletion error rates. This can provide useful information for future applications to current long read sequencing datasets, which also have higher insertion-deletion error rates compared to short-read technologies. Despite differences between the GEMS and Bangladesh datasets, ATLAS identified sub-genus partitions in important taxa previously associated with diarrhea. This improved resolution will provide greater insight into potentially harmful or beneficial organisms.

An opportunity for future research is the integration of the approach embodied in ATLAS with phylogenetic algorithms. Phylogenetic approaches can use the partitions identified by ATLAS to prune the reference tree before attempting to place query sequences on the tree, resulting in higher accuracy with lower computational overhead. In the future, we also plan to identify and investigate cases where ATLAS assignments and phylogenetic classifications disagree in order to identify opportunities for improvements to either alignment-based or phylogenetic approaches. As the wealth of microbiome data increases, greater emphasis is being placed on more accurate taxonomic annotations that currently cannot be obtained using fast, sequence similarity-based methods. ATLAS is the first step in this direction.

## Data Availability Statement

The datasets analyzed for this study can be found in the Human Microbiome Project Data Portal [https://www.hmpdacc.org/HMQCP/] and the GEMS Study of Childhood Diarrheal Disease [http://www.cbcb.umd.edu/datasets/gems-study-diarrheal-disease]. The ATLAS pipeline can be found on GitHub [https://github.com/shahnidhi/outlier_in_BLAST_hits].

## Author Contributions

NS and MP conceived the research project. NS designed and implemented the algorithm, with the help of JSM and MP. NS and JSM analyzed the data. NS, JSM, and MP wrote the manuscript. All authors read and approved the final manuscript.

## Funding

This work was supported in part by the Center for Health-related Informatics and Bioimaging, a Center organized under the MPowering the State Partnership between the University of Maryland Baltimore and College Park campuses. NS, JM, and MP were supported by grants to MP, including grant IIS-1513615 from the NSF.

## Conflict of Interest

The authors declare that the research was conducted in the absence of any commercial or financial relationships that could be construed as a potential conflict of interest.
