# Supplementary material for: Embracing Ambiguity in the Taxonomic Classification of Microbiome Sequencing Data
Source: Front Genet. 2019 Oct 17;10:1022. doi: 10.3389/fgene.2019.01022 (PMC6811648; doi:10.3389/fgene.2019.01022)
Supplement: Supplementary file 1 [file DataSheet_1.docx]

***Supplementary Material***

**
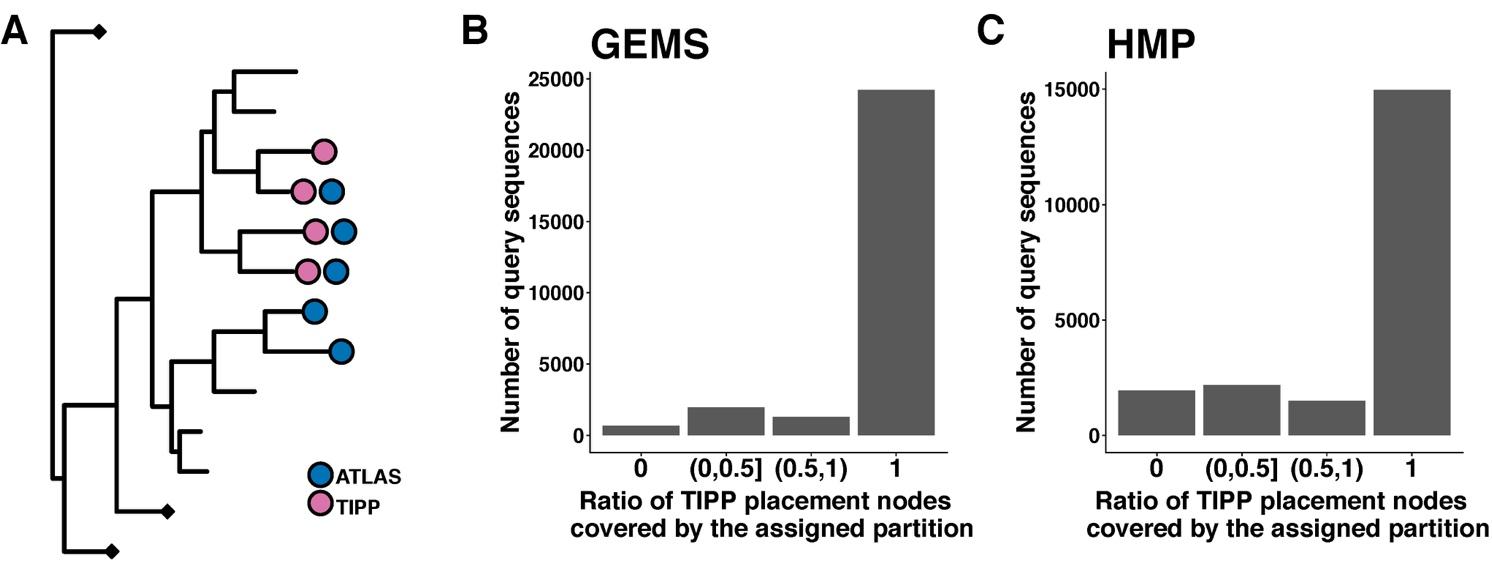
**

**Supplemental Figure 1. ATLAS partitions capture placement nodes identified by TIPP.** (A) An example showing reference database sequences identified by TIPP’s placement and ATLAS’s partition assignment for a query sequence. The ratio of TIPP placement nodes covered by the assigned partition for this query sequence is ¾=0.75. Partitions assigned by ATLAS contain a majority of reference database sequences identified by TIPP’s placement in the (B) GEMS and (C) HMP datasets.

**Supplemental Figure 2. Comparison of ATLAS to other taxonomic annotation methods.** Using a dataset where the ground truth is known, we characterized the performance of different classification methods by several metrics. Both the dataset (sp_ten_16s_v35 ) and metrics used are from Edgar, R. C. (2018). Sequences provided at the TAXXI website (<https://drive5.com/taxxi/doc/index.html>) were split into test and train dataset, such that for all test sequences, the most similar train sequence has given percent identity (horizontal facet for 100, 99, 97, 95). Reported here are (A) raw counts of true positives (TP), true negatives (TN), misclassified sequences (MC), over classified sequences (OC) and under classified sequences (UC). Also shown are (B) classification rates, including accuracy (Acc), true positive rate (TPR), misclassification rate (MCR), over classification rate (OCR), and under classification rate (UCR) for the same dataset.

**
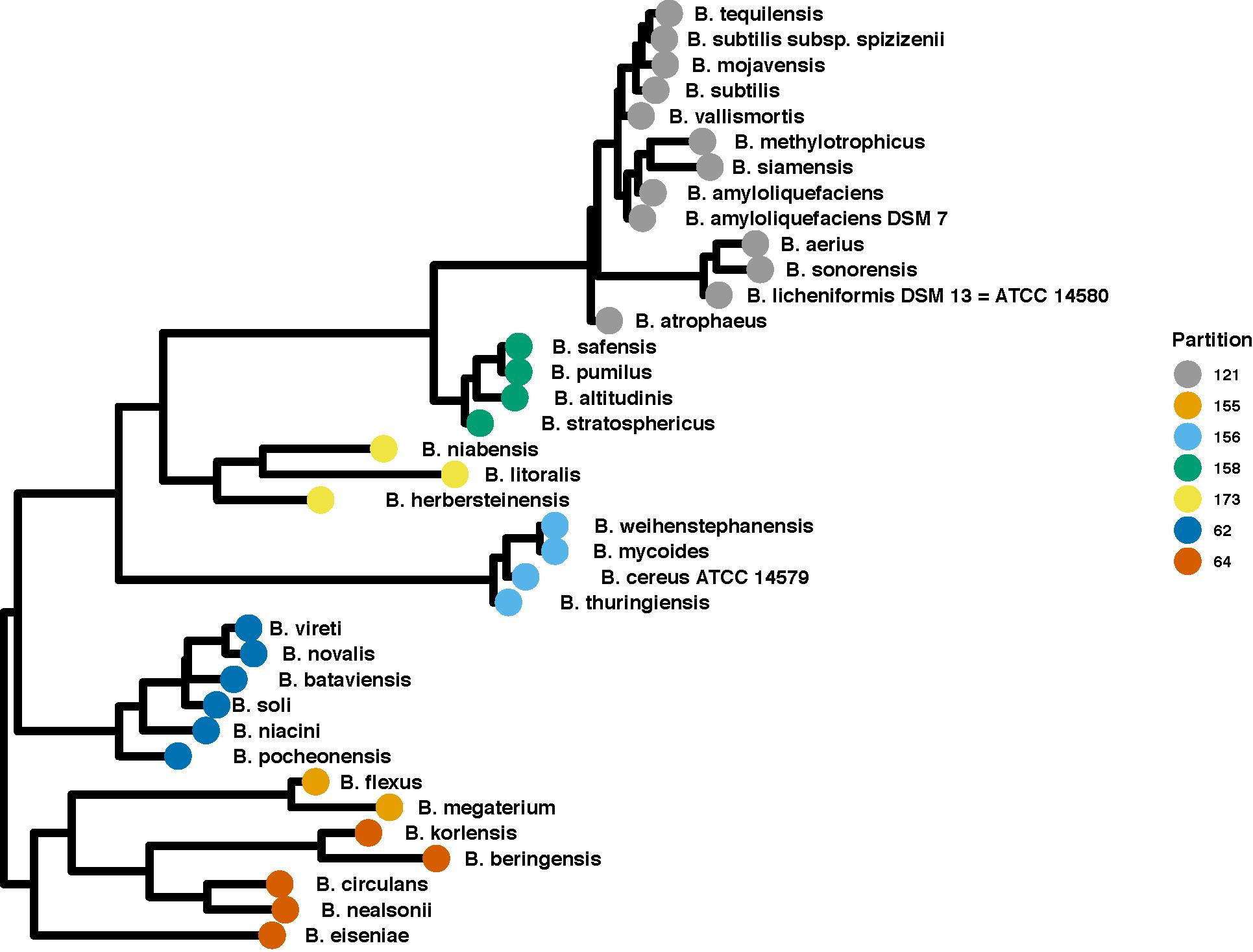
**

**Supplemental Figure 3. Reference database sequences in the sub-genera *Bacillus* partition in HMP samples.** The TIPP reference tree was plotted using ggtree in R. Taxa included in partitions in the HMP dataset are indicated by dots, colored by partition. Branches not identified in our partitions were collapsed for visualization purposes.

**
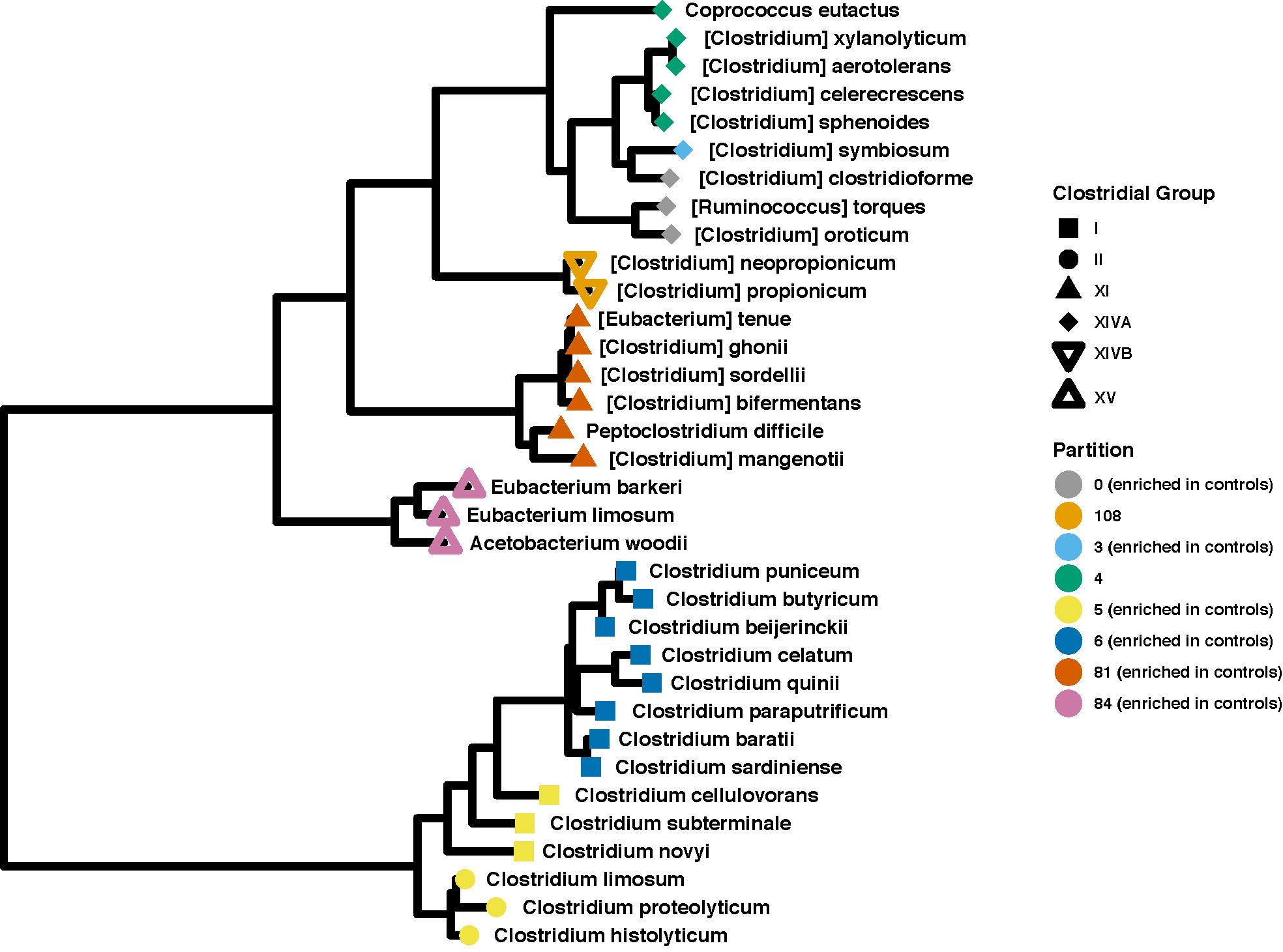
**

**Supplemental Figure 4. Reference database sequences in the Clostridial partition from the GEMS dataset.** The TIPP reference tree was plotted using ggtree in R. Tree labels represent *Clostridia* grouped into partitions in the GEMS dataset, with the node colors representing the different partitions. The points on the tree nodes represent members of *Clostridia* groups identified in (Collins et al., 1994). Branches not identified in our partitions were collapsed for visualization purposes, as were branches of *Clostridia* in the reference tree not grouped by Collins et al. 1994.

**
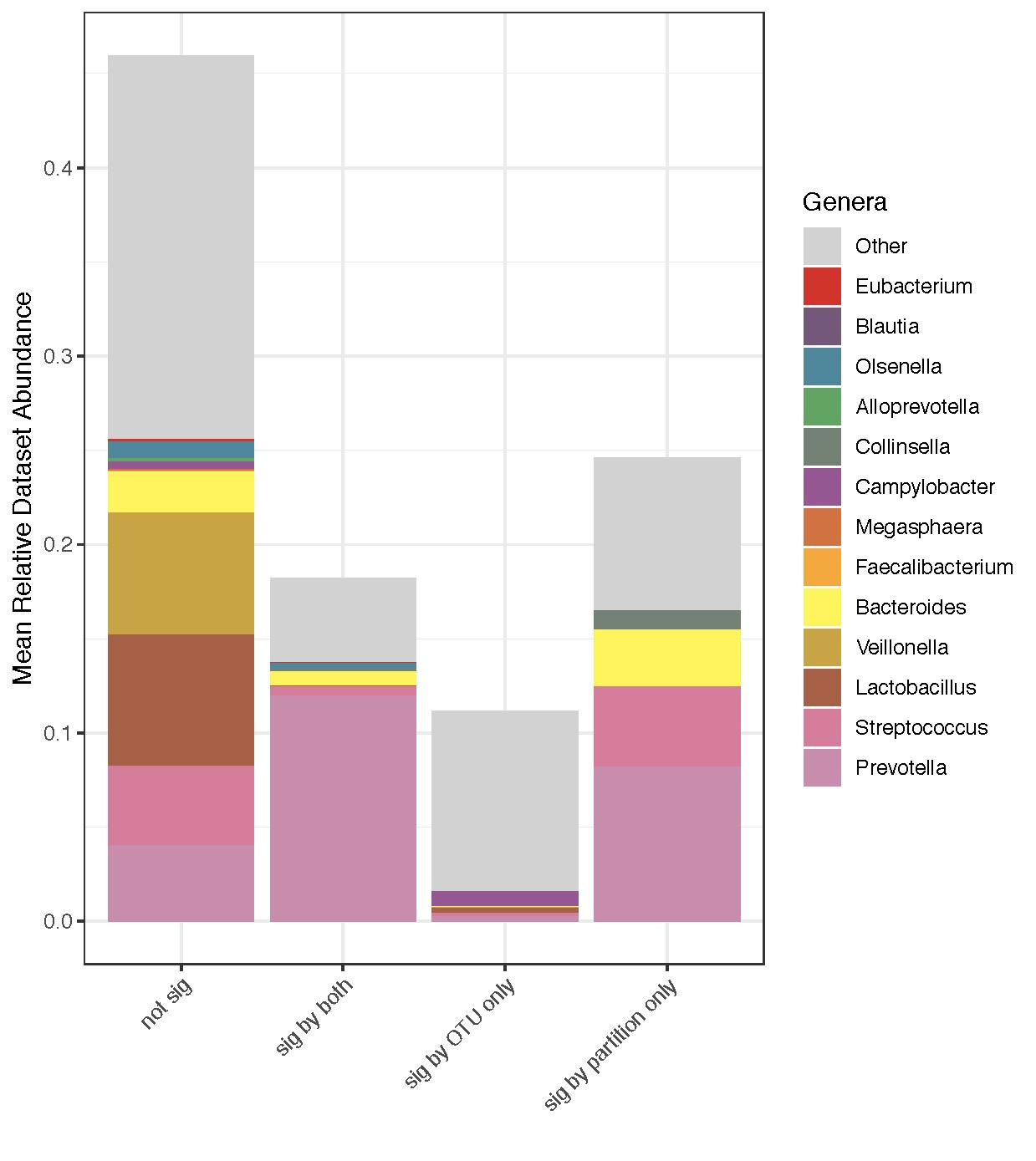
Supplemental Figure 5. Differentially abundant OTUs in the GEMS dataset by genera.** OTUs are grouped by whether they are not significant by either ATLAS partitions or individual OTUs, significant by both ATLAS partitions and individual OTUs, significant by individual OTUs only, or significant by ATLAS partitions only.


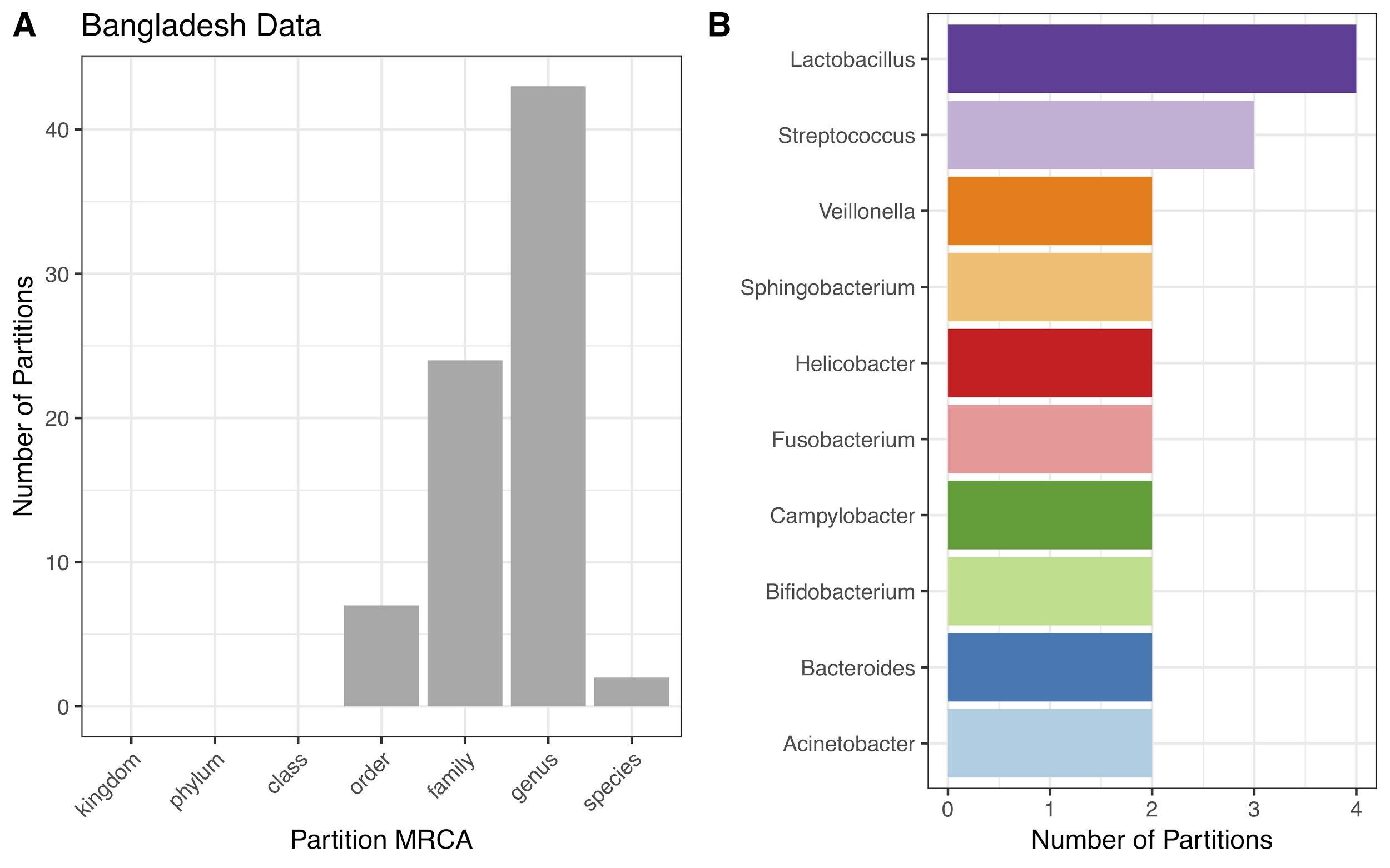


**Supplemental Figure 6. Partitions identified by ATLAS in acute diarrhea samples from Bangladesh.** (A) Most partitions have the most recent common ancestor at the genus level for this dataset. (B) Number of partitions for the most common genera with sub-genus resolution in the Bangladesh dataset.
